# Phenotypic selection on flowering phenology and pollination efficiency traits between Primula populations with different pollinator assemblages

**DOI:** 10.1002/ece3.3258

**Published:** 2017-08-17

**Authors:** Yun Wu, Qing‐Jun Li

**Affiliations:** ^1^ Key Laboratory of Tropical Forest Ecology Xishuangbanna Tropical Botanical Garden Chinese Academy of Sciences Menglun, Mengla County China; ^2^ University of Chinese Academy of Sciences Beijing China; ^3^ Laboratory of Ecology and Evolutionary Biology State Key Laboratory for Conservation and Utilization of Bio‐Resources in Yunnan, Yunnan University Kunming China

**Keywords:** different pollinator assemblages, floral evolution, phenotypic selection, *Primula secundiflora*, spatial variation

## Abstract

Floral traits have largely been attributed to phenotypic selection in plant–pollinator interactions. However, the strength of this link has rarely been ascertained with real pollinators. We conducted pollinator observations and estimated selection through female fitness on flowering phenology and floral traits between two *Primula secundiflora* populations. We quantified pollinator‐mediated selection by subtracting estimates of selection gradients of plants receiving supplemental hand pollination from those of plants receiving open pollination. There was net directional selection for an earlier flowering start date at populations where the dominant pollinators were syrphid flies, and flowering phenology was also subjected to stabilized quadratic selection. However, a later flowering start date was significantly selected at populations where the dominant pollinators were legitimate (normal pollination through the corolla tube entrance) and illegitimate bumblebees (abnormal pollination through nectar robbing hole which located at the corolla tube), and flowering phenology was subjected to disruptive quadratic selection. Wider corolla tube entrance diameter was selected at both populations. Furthermore, the strength of net directional selection on flowering start date and corolla tube entrance diameter was stronger at the population where the dominant pollinators were syrphid flies. Pollinator‐mediated selection explained most of the between‐population variations in the net directional selection on flowering phenology and corolla tube entrance diameter. Our results suggested the important influence of pollinator‐mediated selection on floral evolution. Variations in pollinator assemblages not only resulted in variation in the direction of selection but also the strength of selection on floral traits.

## 1 INTRODUCTION

In angiosperms, plant–pollinator interactions are thought to drive floral evolution, leading to a diversity of floral patterns (reviewed in Harder & Johnson, 2009; Sletvold, Trunschke, Smit, Verbeek, & Ågren, 2016). Pollinator‐mediated selection on floral signals (traits) can be extensive and strong (Schiestl & Johnson, 2013). In generalized pollination systems, different pollinator assemblages and pollinator behaviors may result in spatial and temporal variations in pollinator‐mediated selection among plant populations (Chapurlat, Ågren, & Sletvold, 2015). Studies in path analysis have suggested that variation in selection on floral shape among populations is linked to variation in the composition of local pollinator assemblages (Gómez, Perfectti, Bosch, & Camacho, 2009). In *Aquilegia coerulea*, populations visited by *Sphinx vashti* had longer spurs than populations visited by *Hyles lineate* (Sphingidae). In addition, flowers in populations of *Aquilegia coerulea* with a greater percentage of nectar‐collecting pollinators were not whiter, larger, or with longer spurs than populations visited by few percentages of nectar‐collecting pollinators (Brunet, 2009). Compared with diurnal pollinators in the orchid *Gymnadenia conopsea* population, only nocturnal pollinators selected for longer spurs and mediated stronger selection on number of flowers (Chapurlat et al., 2015). In recent years, there have been an increasing number of studies that use supplemental hand pollination to quantify pollinator‐mediated selection on floral traits (Schiestl & Johnson, 2013; Sletvold & Ågren, 2016; Sletvold, Moritz, & Ågren, 2015; Sletvold et al., 2016). However, it is rare to experimentally quantify pollinator‐mediated selection on floral traits together with supplemental hand pollination and pollinator observations. Using these methods, we can point out the contributions of different pollinators to the direction and strength of selection on floral traits.

Pollinator‐mediated selection can be calculated by comparing the intensity of selection in open‐pollinated and supplemental hand‐pollinated plants (Fishman & Willis, 2008; Sletvold & Ågren, 2010, 2011). If pollinators are exerting selection on floral traits through male and/or female fitness, then selection should be stronger in the naturally pollinated treatment (Bartkowska & Johnston, 2012).

In this study, we experimentally quantified pollinator‐mediated selection on flowering phenology, floral display, and pollination efficiency traits through female fitness and also pointed out the dominant pollinators at two *Primula secundiflora* populations. *P. secundiflora* has a generalized pollination system and is mostly visited by bumblebees and syrphid flies (Zhu, Jiang, Li, Zhang, & Li, 2015). After initial observations, we found that there were differences in the pollinator assemblages between our two studied populations. Because *P. secundiflora* is a distylous, self‐ and intra‐incompatible herb, reproductive success of this species is dependent on pollinators. Different pollinator assemblages are likely to result in variations in the pollinator‐mediated selection on flowering phenology and floral traits. Here, we tested variation in the net directional selection and pollinator‐mediated selection on flowering phenology and floral traits between two *P. secundiflora* populations. We specifically ask: (1) whether selection on flowering phenology, floral display, and pollination efficiency traits varies between populations with different pollinator assemblages; (2) whether this variation can be explained by variation in pollinator‐mediated selection.

## 2 MATERIALS AND METHODS

### 2.1 Study species and sites

*Primula secundiflora* is a distylous [long style and short anther phenotype (L‐morph), short style and long anther phenotype (S‐morph)], self‐ and intramorph‐incompatible perennial herb that is widely distributed throughout the alpine regions of southwest China. It produces leaves in a basal rosette and normally has 3–43 flowers in a single umbel. Its flowering period is from May to August, and the fruiting period is from August to September. Our field experiments were performed at the Bigu Tianchi Scenic Spot (BGTC, 27°37′53.209″N, 99°41′13.544″E, 3605 m.a.s.l.) population and the Potatso National Park (PNP, 27°47′55.541″N, 99°54′35.766″E, 3433 m.a.s.l.) population in Shangri‐La Country, Yunnan Province, China. The two populations were separated by 20 km. In both of our study sites, *P. secundiflora* was the dominant species, with thousands of individuals being present.

### 2.2 Pollinators

In 2016, we conducted pollinator observations at the two populations. At each population, we set two or three 2 × 2 m plots. There were between 120 and 150 *P. secundiflora* individuals in each plot. Pollinators were recorded during a series of 30‐min sessions from 0830 to 1,830 over three sunny days at each population. We recorded a plant visitor as a pollinator if it touched the sexual organs of the flower during its visit. For each observation, we recorded the types, numbers, and behaviors of pollinators. We used the mean numbers of pollinators/plot/hour/individual as a proxy in our analysis.

### 2.3 Field experiment

To quantify pollinator‐mediated selection on flowering phenology, floral display, and pollination efficiency traits, we conducted experimental hand pollination at both populations during May–August 2016. Before flowering, plants with flower buds were randomly chosen (including both S‐ and L‐morph plants) and individually tagged in each population. We randomly marked 137 and 120 plants at BGTC and PNP populations, respectively. The study populations were visited twice a week throughout the flowering period, and during each visit, all open flowers on plants in the supplemental hand pollination treatment were pollinated by hand with cross‐pollen from other plants which were located at least 10 meters away from the target. All flowers received supplemental hand pollination at least once. For the pollination of the S‐morph flowers, we followed the methods of Zhu et al. (2015) and punctured the corolla tube near the stigma and brushed dehiscing anthers across receptive stigmas through the hole using tweezers. This method did not influence the seed production.

### 2.4 Measured traits and female fitness

**Figure 1:**
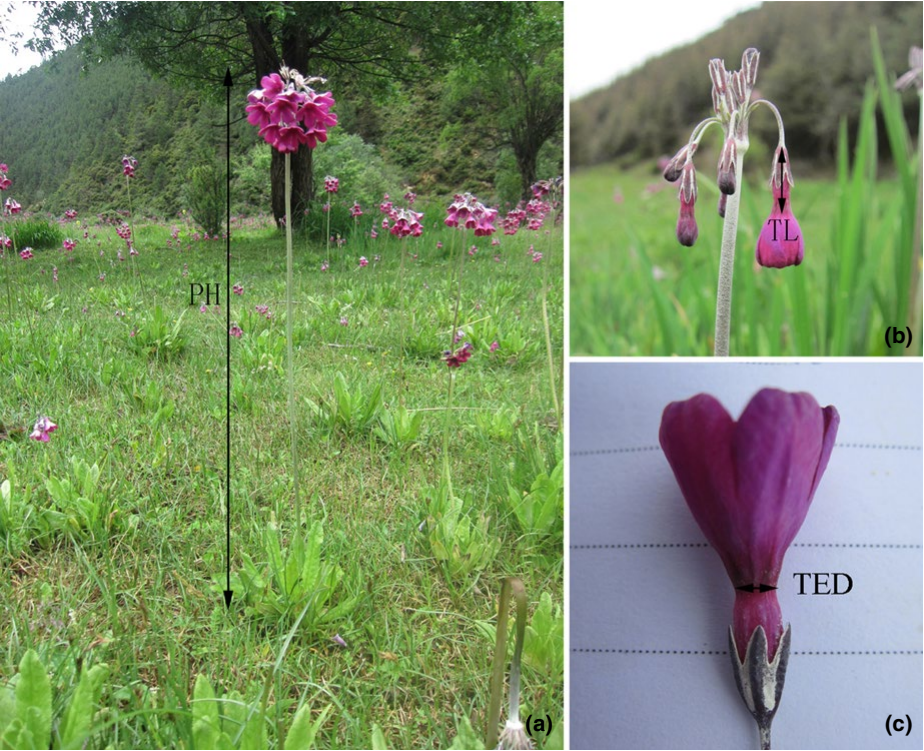
Illustration of the phenotypic traits measured in this study on *Primula secundiflora*. (a) PH, plant height; (b) TL, corolla tube length; (c) TED, corolla tube entrance diameter

We recorded the flowering start date (Julian day) for each individual as the day on which the first flower opened. At the onset of flowering, we measured plant height of each individual included in the experiment (distance from the ground to the topmost flower, Figure 1a). On the first five open flowers of each individual, we measured corolla tube length (distance from the corolla tube entrance to corolla tube bottom, Figure 1b) and corolla tube entrance diameter (Figure 1c) to the nearest 0.01 mm with digital calipers. Number of flowers per plant was recorded at the end of the flowering period.

To quantify female reproductive success, we recorded the number of fruits at maturation, and we collected all fruits from each plant to determine viable seeds per fruit. For each individual, we estimated female fitness as the total viable seeds. We quantified pollen limitation (PL) for each population as 1‐(mean female fitness of open‐pollinated control plants/mean female fitness of hand‐pollinated plants).

### 2.5 Statistical analyses

Basically, there were no significant differences (*p* > .05) in the floral traits and reproductive performance between morphs (L and S) at either population (Table S1). Based on this information, we combined the L‐ and S‐morph data in the statistical analyses in this study. Effects of pollination treatments (control vs. supplemental hand pollination) and populations on flowering phenology, floral traits, and reproductive performance were tested with two‐way ANOVA. In order to improve normality of data analysis, all data were log_10_‐transformed before the analyses.

Following the methods of Lande and Arnold (1983), we used multiple regression analysis to estimate the direction and strength of net directional selection and pollinator‐mediated selection. In these regression models, we used the relative female fitness (individual female fitness/mean female fitness) and the standardized traits (with a mean of 0 and a variance of 1) to be as the response variable and explanatory variables, respectively. In addition, the relative female fitness and standardized traits were estimated separately for each treatment and each population. We estimated selection gradients separately for each treatment and each population. Using the multiple linear regression models, we quantified the net directional selection gradients (β_i_). We estimated the nonlinear selection gradients (γ_ii_) using the quadratic terms of multiple nonlinear regression models. In order to limit model complexity, we did not include the cross‐product terms in the regression models. In order to obtain the real selection pressure, we doubled the nonlinear selection gradient coefficients (γ_ii_) from the quadratic regression model (Stinchcombe, Agrawal, Hohenlohe, Arnold, & Blows, 2008). To test multicollinearity in the regression models, we calculated variance inflation factors (VIFs) for the linear terms and quadratic terms. All VIFs were <3.2, indicating no problem of multicollinearity.

We used ANCOVA to determine whether net directional selection gradients varied between populations using the data from the open‐pollinated control treatment (the first model). In addition, we used ANCOVA to determine whether pollinator‐mediated selection gradients varied between populations using the data from both open‐pollinated control and supplemental hand pollination treatments (the second model). In the first model, we used the relative female fitness to be as response variable, and used five standardized traits (flowering start date, plant height, number of flowers, corolla tube length, and corolla tube entrance diameter), population and trait × population interactions to be as the explanatory variables. In this model, a significant trait × population interaction demonstrated that net directional selection varied between populations. In the second model, we used the relative female fitness to be as the response variable and used five standardized traits (the same as above), pollination (open‐pollinated control vs. supplemental hand pollination), population, trait × pollination, trait × population, and trait × pollination × population interactions to be as the explanatory variables. In this model, a significant trait × pollination × population interaction demonstrated that pollinator‐mediated selection varied between populations. Significant three‐way interactions were detected in these models; we further examined the effect of pollination treatment on selection gradients for each population and used the trait × pollination interaction term to determine whether pollinator‐mediated selection on floral traits was significant. To quantify pollinator‐mediated selection, we subtracted for each trait the estimated selection gradient for plants receiving supplemental hand pollination (β_HP_ or γ_HP_) from the estimate obtained for open‐pollinated controls (β_C_ or γ_C_), Δβ_poll_ = β_C_ − β_HP_, or Δγ_poll_ = γ_C_ − γ_HP_ (Chapurlat et al., 2015). Pollinator‐mediated linear (Δβ_poll_) and quadratic (Δγ_poll_) selections on each floral trait were quantified separately for each population. All analyses were performed with R 3.2.3. We used Excel (2007) to generate graphs.

## 3 RESULTS

### 3.1 Pollinator assemblages

**Figure 2:**
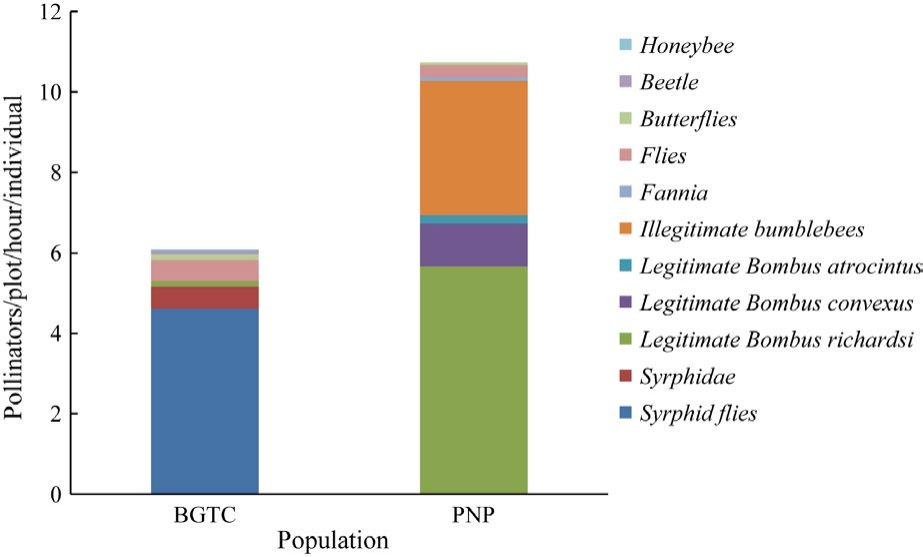
Pollinator observations conducted in two *Primula secundiflora* populations. Legitimate *Bombus atrocintus*, legitimate *Bombus convexus,* and legitimate *Bombus richardsi* meant pollinators normally pollinated flowers through corolla tube entrance. Illegitimate bumblebees meant pollinators abnormally pollinated flowers through nectar robbing hole which located at the corolla tube

We carried out a total of 16.5 hr of observations in each population. At BGTC population, syrphid flies were the most abundant pollinators (Figure 2), accounting for 75.62% of total pollinators. However, legitimate bumblebees (normal pollination through the corolla tube entrance) and illegitimate bumblebees (abnormal pollination through nectar robbing hole which located at the corolla tube) were the most dominant pollinators at PNP population. Legitimate bumblebees accounted for 64.69% of total pollinators, and illegitimate bumblebees accounted for 31.07% of total pollinators. Legitimate bumblebees included *Bombus richardsi*,* B. convexus*, and *B. atrocintus* (Figure 2). Illegitimate bumblebees included some *B. richardsi*,* B. lucorum,* and few *B. atrocintus*. Illegitimate bumblebees consumed nectar by biting a hole in the corolla tube or using a hole already bitten in the tube. Illegitimate bumblebees increased the pollen flow between low reproductive organs (the anthers of L‐morph flowers and the stigmas of the S‐morph flowers) and pollinated the S‐morph flowers in *P. secundiflora* (own data, unpublished).

### 3.2 Floral traits and phenotypic correlations

Flowering start date, plant height, number of flowers, and corolla tube entrance diameter varied between populations (Tables 1 and S2; Fig. S1). At BGTC population, plants that were taller consist of more flowers and earlier flowering start date. At PNP population, plants started to flower later, lower plant vegetation, and produced lesser flowers. At both populations, most floral traits were positively correlated with each other, except the flowering start date at BGTC population, which was negatively correlated with the other traits (Table S3).

**Table 1:** Flowering phenology, floral traits, and reproductive success (mean ± *SD*) for plants receiving open‐pollinated control treatment (C) and supplemental hand pollination (HP) plants in the *Primula secundiflora* populations. Sample sizes *n* are given in these orders

Trait, by study site	C (*n* = 66/64)	HP (*n* = 71/56)
Flowering start date (Julian day)		
BGTC	149 ± 1.3	149 ± 1.6
PNP	166 ± 1.5	166 ± 1.3
Plant height (cm)		
BGTC	58.83 ± 7.74	61.35 ± 7.05
PNP	44.60 ± 7.06	43.26 ± 6.36
Number of flowers		
BGTC	23.9 ± 8.2	28.9 ± 9.0
PNP	21.6 ± 5.9	17.8 ± 4.9
Corolla tube length (mm)		
BGTC	10.10 ± 0.68	10.38 ± 0.74
PNP	10.20 ± 0.82	10.12 ± 0.88
Corolla tube entrance diameter (mm)		
BGTC	3.92 ± 0.50	3.81 ± 0.44
PNP	3.65 ± 0.55	3.65 ± 0.60
Number of fruits		
BGTC	6.8 ± 3.8	14.5 ± 5.1
PNP	12.4 ± 6.6	14.1 ± 4.4
Viable seeds per fruit		
BGTC	78.4 ± 36.5	144.6 ± 27.3
PNP	121.4 ± 29.6	131.4 ± 20.8
Female fitness (viable total seeds per individual)		
BGTC	565 ± 491	2,158 ± 1,005
PNP	1,608 ± 1,056	1,893 ± 748

BGTC, Bigu Tianchi Scenic Spot; PNP, Potatso National Park.

### 3.3 Reproductive success and pollen limitation

Plants produced more fruits, more viable seeds per fruit, and more female fitness (total viable seeds per individual) of open pollination at the PNP population than those at the BGTC population (Table 1). Supplemental hand pollination significantly increased fruit production, viable seeds per fruit, and female fitness (Tables 1 and S2). Fruit production, viable seeds per fruit, and female fitness were pollen‐limited at both populations. Pollen limitation of female fitness varied between populations (PL = 0.738 and 0.151 at BGTC and PNP populations, respectively; *F*_1, 256_ =55.776, *p* < .001; Table S2). Pollen limitation of female fitness was higher at the BGTC population than it at the PNP population.

### 3.4 Phenotypic selection

**Figure 3:**
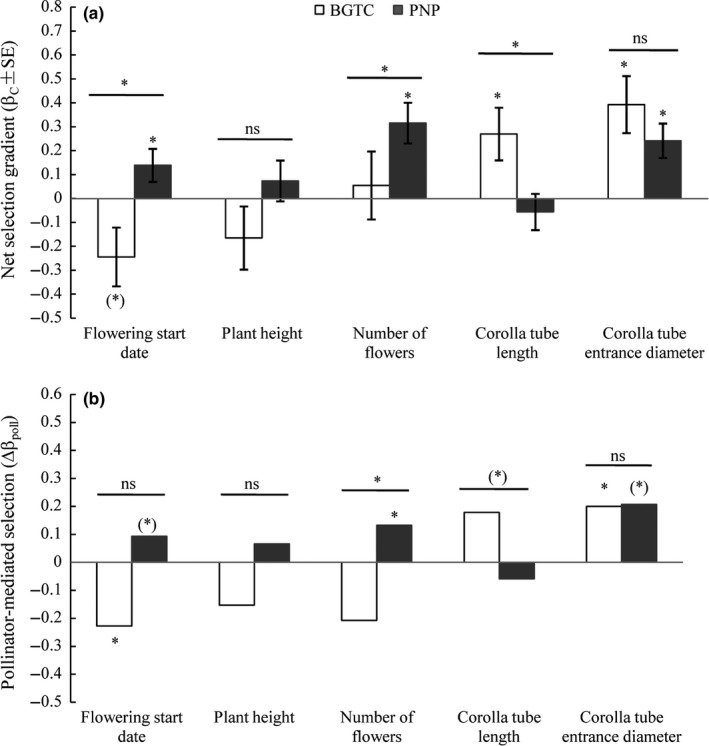
Linear selection gradients among open‐pollinated control plants (a) and pollinator‐mediated selection gradients (b) on flowering start date, plant height, number of flowers, corolla tube length, and corolla tube entrance diameter in the two *Primula secundiflora* populations. Symbols above individual bars indicated the level of significance of the selection gradient. Symbols above the lines spanning several gradients in the (a) and (b) graphs show whether ANCOVA indicated significant variation between populations in net directional selection gradient (significant trait × population interaction) and pollinator‐mediated selection gradient (significant trait × pollination × population). *, *p* < .05; (*), *p* < .1; ns, *p* > .1

Net directional selection on flowering start date, number of flowers, and corolla tube length varied between populations (Figures 3a and S1, Tables 2 and S4). At BGTC population, earlier flowering start date was statistically significant selected (β_C_ = −0.244 ± 0.123; Figure 3a, Table 2); however, later flowering start date was significant selected at PNP population (β_C_ = 0.139 ± 0.069; Figure 3a, Table 2). Plants tended to produce more flowers at PNP population; however, net directional selection on number of flowers at BGTC population was not statistically significant. Comparing with BGTC population (β_C_ = 0.055 ± 0.142; Figure 3a, Table 2), the strength (the absolute selection gradients) of net directional selection on number of flowers was stronger at PNP population (β_C_ = 0.316 ± 0.085; Figure 3a, Table 2). Longer corolla tube was statistically significant selected at BGTC population (β_C_ = 0.27 ± 0.11, *p *=* *.017). Finally, there was statistically significant net directional selection for wider corolla tube entrance diameter at both populations (β_C_ = 0.393 ± 0.119 at BGTC population and β_C_ = 0.242 ± 0.072 at PNP population; Figure 3a, Table 2). The strength of net directional selection on corolla tube entrance diameter was stronger at BGTC population than that at PNP population.

**Table 2:** Linear (β_*i*_ ± *SE*) and quadratic selective gradients (γ_*ii*_ ± *SE*) and associated *p*‐values among open‐pollinated control plants (C) and hand‐pollinated plants (HP) in the two *Primula secundiflora* populations. Pollinator‐mediated linear (Δβ_poll_ = β_C_ − β_HP_) and quadratic (Δγ_poll_ = γ_C_ – γ_HP_) selections and *p*‐value association with the trait × pollination treatment interactions in ANCOVAs conducted separately for each population are also given

	C				HP				Pollinator‐mediated selection			
	β_i_ ± *SE*	*p*	γ_ii_ ± *SE*	*p*	β_i_ ± *SE*	*p*	γ_ii_ ± *SE*	*p*	Δβ_poll_	*p*	Δγ_poll_	*p*
BGTC, PL = 0.738												
Flowering start date	**−0.244 ± 0.123**	**.053**	**−0.321 ± 0.082**	**.056**	**−**0.016 ± 0.047	.732	**−**0.018 ± 0.039	.822	**−0.228**	**.047**	**−0.303**	**.029**
Plant height	**−**0.165 ± 0.132	.218	0.115 ± 0.086	.506	**−**0.012 ± 0.053	.817	**0.119 ± 0.028**	**.035**	**−**0.153	.340	**−**0.004	.851
Number of flowers	0.055 ± 0.142	.701	**−0.316 ± 0.080**	**.052**	**0.263 ± 0.045**	**<.001**	**−**0.029 ± 0.031	.634	**−**0.208	.113	**−0.287**	**.045**
Corolla tube length	**0.270 ± 0.110**	**.017**	0.216 ± 0.076	.161	0.092 ± 0.054	.096	0.137 ± 0.043	.119	0.178	.207	0.079	.071
Corolla tube entrance diameter	**0.393 ± 0.119**	**.002**	0.221 ± 0.103	.287	**0.193 ± 0.057**	**.001**	0.007 ± 0.041	.928	**0.200**	**.028**	0.214	.356
PNP, PL=0.151												
Flowering start date	**0.139 ± 0.069**	**.050**	**0.331 ± 0.064**	**.012**	0.046 ± 0.060	.445	0.067 ± 0.043	.445	**0.093**	**.053**	0.264	.145
Plant height	0.074 ± 0.085	.390	0.005 ± 0.043	.956	0.008 ± 0.068	.913	**0.149 ± 0.034**	**.036**	0.066	.476	**−**0.144	.252
Number of flowers	**0.316 ± 0.085**	**.001**	**−**0.079 ± 0.052	.447	**0.184 ± 0.062**	**.005**	**−**0.146 ± 0.041	.079	**0.132**	**.036**	0.067	.850
Corolla tube length	**−**0.056 ± 0.076	.465	**−**0.072 ± 0.048	.461	0.003 ± 0.071	.962	**−**0.032 ± 0.040	.694	**−**0.059	.381	**−**0.040	.874
Corolla tube entrance diameter	**0.242 ± 0.072**	**.002**	0.140 ± 0.070	.320	0.035 ± 0.067	.603	**−0.246 ± 0.060**	**.046**	**0.207**	**.058**	**0.386**	**.049**

BGTC, Bigu Tianchi Scenic Spot; PNP, Potatso National Park.

PL is the pollen limitation of female fitness estimated as 1‐(mean female fitness of open‐pollinated control plants/mean female fitness of hand‐pollinated plants).

Significant selection estimates and their *p*‐values are indicated in bold.

In open‐pollinated control treatment plants, significant quadratic selection gradients of flowering start date and number of flowers were detected at least in one population. At BGTC, flowering start date (γ_C_ = −0.321 ± 0.082, *p *=* *.056; Table 2) and number of flowers (γ_C_ = −0.316 ± 0.080, *p *=* *.052; Table 2) were subjected to marginal significant quadratic gradients. In addition, flowering start date (γ_C_ = 0.331 ± 0.064, *p *=* *.012; Table 2) was subjected to significant quadratic gradient at PNP population. These results indicated that flowering phenology was subjected to stabilizing selection in the population where the dominant pollinator was syrphid flies, whereas flowering phenology was subjected to disruptive selection in the population where the dominant pollinators were legitimate and illegitimate bumblebees.

### 3.5 Pollinator‐mediated selection

**Figure 4:**
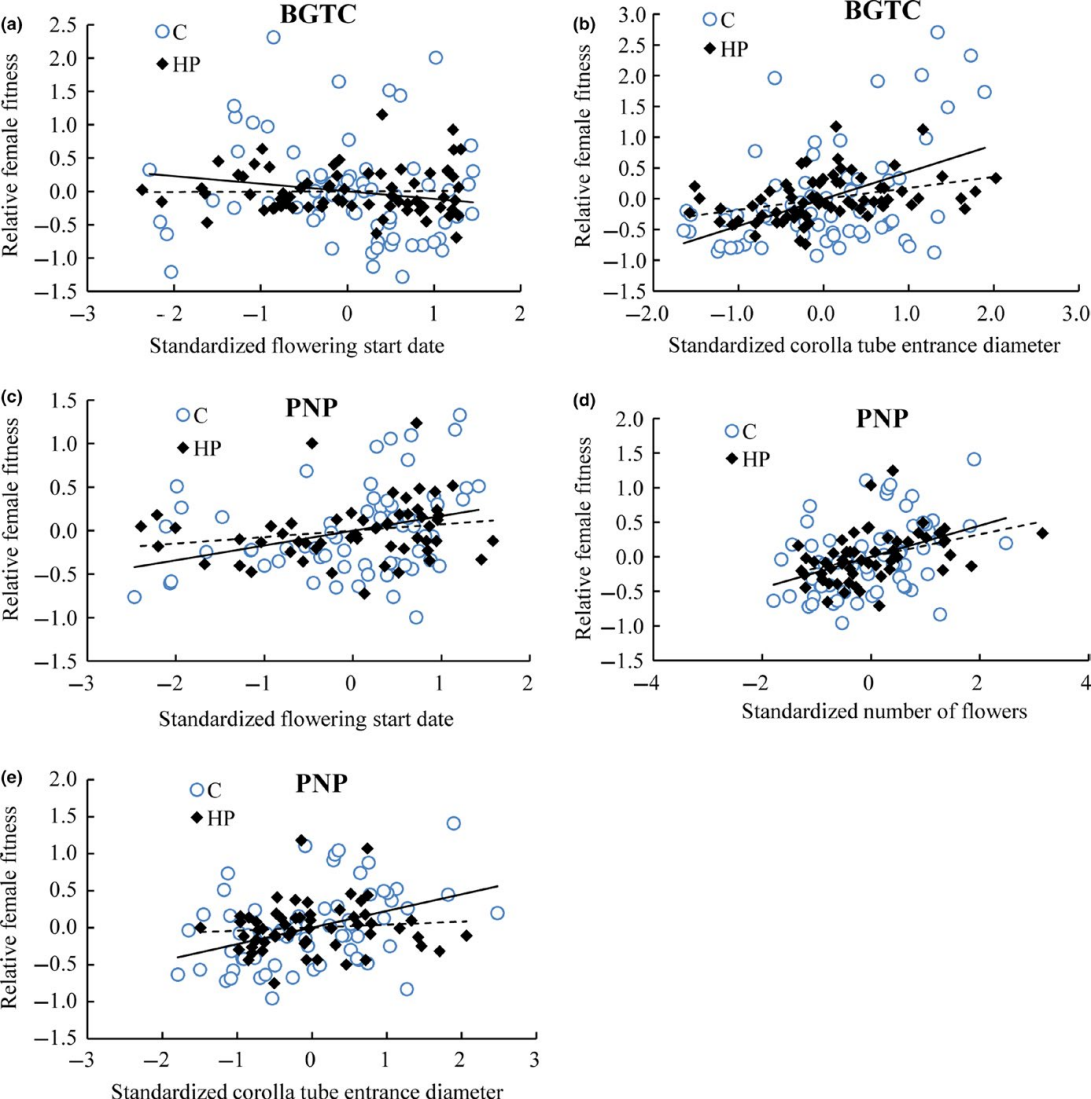
Standardized linear phenotypic selection gradients for flowering start date, number of flowers, and corolla tube entrance diameter in open‐pollinated plants (C, open circles, solid line) and in supplemental hand pollination plants (HP, closed circles, dashed line) at Bigu Tianchi Scenic Spot (BGTC) (a, b) and Potatso National Park (PNP) populations (c–e). Selection gradients were illustrated with added‐variable plots, in which the residuals from a linear regression model of relative female fitness on all traits except the focal trait are plotted against the residuals from a regression model of the focal trait on the other traits

Pollinator mediated significant selection on flowering phenology, number of flowers, and corolla tube entrance diameter (Table 2). Much of the between‐population variations in net directional selection on these three traits could be explained by the variations in pollinator‐mediated selection (Figure 3b; Table 2). At BGTC population, pollinator‐mediated selection for an earlier flowering start date (Δβ_poll_ = −0.228) accounted for 93.4% [(−0.228)/(−0.244) × 100%] of the observed net directional selection (Figures 3b and 4a; Table 2). At PNP population, pollinator‐mediated selection for a later flowering start date (Δβ_poll_ = 0.093, *p *=* *.053) accounted for 66.91% [(0.093/0.139) × 100%] of the observed net directional selection (Figures 3b and 4c; Table 2) although it was only detected at marginal significance. At PNP population, the significant pollinator‐mediated selection for more flowers (Δβ_poll_ = 0.132) accounted for 41.8% of the observed net directional selection (Figures 3b and 4d; Table 2). Pollinator‐mediated selection (Δβ_poll_ = 0.2 and Δβ_poll_ = 0.207 at BGTC and PNP population; Figures 3b and 4b,e; Table 2) accounted for 50.9% and 85.5% of observed net directional selection on corolla tube entrance diameter at BGTC and PNP population.

Pollinators contributed significantly to quadratic selection, but the strength (the absolute selection gradients) varied between populations (Table 2). At BGTC population, pollinator‐mediated selection (Δγ_poll_ = −0.303) could explain 94.4% of the quadratic selection on flowering start date (Table 2). Significant pollinator‐mediated selection (Δγ_poll_ = −0.287) explained 90.8% of the quadratic selection on number of flowers at BGTC population (Table 2). At PNP population, significant pollinator‐mediated selection (Δγ_poll_ = 0.386) could explain all of the quadratic selection on corolla tube entrance diameter (Table 2).

## 4 DISCUSSION

Ascertaining the causes of variation in phenotypic selection on floral traits among open‐pollinated populations is key to understanding the process of adaptive differentiation (MacColl, 2011). Basically, dominant pollinator, which is always thought to be the most important biotic agent for pollination, can drive variations in phenotypic selection on flowering time (Sandring & Ågren, 2009), floral display (e.g., flower production and flower color) (Carlson & Holsinger, 2010; Chapurlat et al., 2015), and pollination efficiency (e.g., corolla tube length and spur length) (Boberg et al., 2014; Paudel et al., 2016) traits. In this study, variation in the net directional selection on flowering phenology and pollination efficiency (corolla tube entrance diameter) traits between two *P. secundiflora* populations was experimentally demonstrated. Corresponding to the variation in net directional selection, variation in pollinator‐mediated selection on these two traits between populations was also detected. In addition, observed variations in net directional selection on these two traits between populations could be mostly attributed to mediations by different pollinators (bumblebees and syrphid flies).

In generalized pollination systems, pollinator assemblages often vary across the distributional range of plant species, and this can potentially cause variation in selection on flowering phenology (Ehrlén, 2015; Gómez et al., 2009). This hypothesis has been supported in the orchid species, *Dactylorhiza lapponica* (Sletvold, Grindeland, & Ågren, 2010). In our study, we observed different pollinator assemblages between two *P. secundiflora* populations. Furthermore, variation in net directional selection on flowering start date between populations was also detected. Syrphid flies were more abundant in the earlier flowering period at BGTC population, where there was marginally significant selection for earlier flowering start date, and pollinator‐mediated selection could explain most of the observed net directional selection on this trait. In *Penstemon digitalis*, there is significant selection for an earlier flowering date (Parachnowtisch, Raguso, & Kessler, 2012). Moreover, using both ordinary and phylogenetic meta‐analysis, researchers have suggested that selection on alpine plants favored early flowering, resulting in more opportunities to ensure reproductive success (Munguía‐Rosas, Ollerton, Parra‐Tabla, & De‐Nova, 2011). In addition, the significant pollinator‐mediated quadratic selection on flowering start date also indicated the important influence of syrphid flies on this trait at BGTC population. However, in our study, the PNP population experienced significant net directional selection for a later flowering start date and pollinator‐mediated selection explained most of the observed selection. At this population, the dominant pollinators (legitimate and illegitimate bumblebees) were more abundant in the middle of flowering period. This is consistent with studies on *Gymnadenia conopsea* populations (Chapurlat et al., 2015), where pollinator abundance increases during the flowering period, resulting in pollinator‐mediated selection for later flowering. Consequently, differences in phenotypic selection on flowering start date between two *P. secundiflora* populations could be attributed to interactions with pollinators. These patterns of flowering phenology can provide more opportunities for pollination in the *P. secundiflora* populations and then ensure reproductive success in this plant species.

Accurate structures of angiosperm flowers are thought to be shaped by dominant pollinators to improve the efficiency of pollination (reviewed in Boberg et al., 2014; Harder & Johnson, 2009). In the *Ipomopsis aggregata* populations, wider flower width resulted in increasing proportion of available pollen removal and was selected by the dominant pollinators, hummingbirds (Campell, Waser, & Price, 1996). Likewise, pollinator‐mediated selection for wider corolla tubes was also detected in the distylous plant *Primula farinose* (Vanhoenacker, Toräng, Ågren, & Ehrlén, 2010). In this study, we also tested significant net directional selection for wider corolla tube entrance diameter at both populations, and much selection on this trait could be attributed to interactions with pollinators. Consistent pollinator‐mediated selection for wider corolla tube entrance diameter could improve the mechanical fit between the flowers and pollinators in this plant, then result in higher pollination efficiency. Specifically, wider corolla entrance diameter was advantageous for pollinators (bumblebees and syrphid flies) to touch the sexual organs of the flower, and then increased the pollen removal and receipt in both *P. secundiflora* populations.

The strength of phenotypic selection on floral traits through female function is expected to increase with increasing pollen limitation (Bartkowska & Johnston, 2015; Benkman, 2013). Experimental reduction in plant–pollinator interaction intensity (increasing pollen limitation) increases the strength of selection on plant height, corolla size, and spur length in the orchid species, *Gymnadenia conopsea* (Sletvold & Ågren, 2016). Besides studies in one orchid species, researchers also test similar results among 12 other orchid species (Trunschke, Sletvold, & Ågren, 2017). Our results also support this hypothesis on several floral traits. In the present study, there was stronger pollen limitation of *P. secundiflora* at BGTC population than that at PNP population. Consequently, the strength (the absolute selection gradient) of net directional selection on flowering start date and corolla tube entrance diameter at BGTC population was stronger than that at the PNP population. These results may suggest the functional relationship between these two traits and the degree of pollen limitation. In order to clarify this relationship, manipulative experiments are needed in future studies.

As previous studies have suggested, an increase in the number of flowers is always selected because of the upper limit to seed production and attractiveness to pollinators (Grindeland, Sletvold, & Ims, 2005; Mitchell, Karron, Holmquist, & Bell, 2004; Sandring & Ågren, 2009). Selection on corolla tube or spur length can be attributed to interactions with local pollinators (Huang, Wang, & Sun, 2016), for example, coevolutionary elaboration between corolla tube length of *Roscoea purpurea* and its local pollinators (Paudel et al., 2015, 2016). However, our results support these hypotheses at only one of our studied populations. More flower production was selected at PNP population, and pollinator‐mediated selection mostly accounted for this. In contrast, there was no net directional but stabilized selection on number of flowers at the BGTC population. These results may suggest that more flower production is attractive to bumblebees, whereas it is not sensitive to syrphid flies. Furthermore, these may also suggest that flower production of *P. secundiflora* in the BGTC population is optimal to attract pollinators and ensure reproductive success. Although significant net directional selection for longer corolla tube length was detected at BGTC population, pollinator‐mediated selection on this trait was not detected. This may suggest that selection on corolla tube length has been selected by other agents, such as herbivores (Parachnowitsch & Caruso, 2008; Sletvold et al., 2015). Furthermore, flower production (floral display trait) and corolla tube length (floral pollination efficiency trait) may be correlationally selected by dominant pollinators, because of an additive effect of these two traits to reproductive success (Chapurlat et al., 2015).

Field experiments with *P. farinosa* suggest pollinator‐mediated selection for taller plant height (Ågren, Fortunel, & Ehrlén, 2006; Ehrlén, Käck, & Ågren, 2002). However, our results do not support this hypothesis. There was no significant selection on plant height at either population. These results indicate that bumblebees and syrphid flies are not sensitive to this trait at our studied populations. In addition, it is possible that plant height may be selected by other agents, such as resource limitation, herbivores, or vegetation context (Ågren, Hellström, Toräng, & Ehrlén, 2013; Sletvold, Grindeland, & Ågren, 2013; Sletvold, Tye, & Ågren, 2017).

In summary, an earlier flowering start date was selected at the population where the dominant pollinators were syrphid flies, whereas at the population where the dominant pollinators were legitimate and illegitimate bumblebees, a later flowering start date was selected. Wider corolla tube entrance diameter was selected at both populations. Furthermore, the strength of net directional selection on flowering start date and corolla tube entrance diameter was stronger at the population where the dominant pollinators were syrphid flies. Pollinator‐mediated selection explained most of the between‐population variations in the net directional selection on these two traits. Our results suggest the important influence of pollinator‐mediated selection on floral evolution. Variations in pollinator assemblages not only result in variation in the direction of selection but also the strength of selection on floral traits. Intriguingly, there are two different pollinators’ foraging behaviors (legitimate and illegitimate bumblebees) at PNP population. A full understanding of the role of legitimate and illegitimate pollinators on differentiation and maintenance of floral traits requires more experimental studies. Finally, patterns of pollinator‐mediated selection on floral traits may differ between male and female fitness (Benitez‐Vieyra, Medina, Glinos, & Cocucci, 2006; Hodgins & Barrett, 2008). In order to comprehensively explore plant–pollinator interactions, it is advisable to pay more attention to both male and female functions in future studies.

## CONFLICT OF INTEREST

None declared.

## AUTHOR CONTRIBUTIONS

Yun Wu and Qing‐Jun Li designed the experiment. Yun Wu conducted the experiment, statistically analyzed the data, and prepared this manuscript.
