# Supplementary figures and images for: Phenotypic selection on flowering phenology and pollination efficiency traits between Primula populations with different pollinator assemblages
Source: Ecol Evol. 2017 Aug 17;7(19):7599–608. doi: 10.1002/ece3.3258 (PMC5632619; doi:10.1002/ece3.3258)

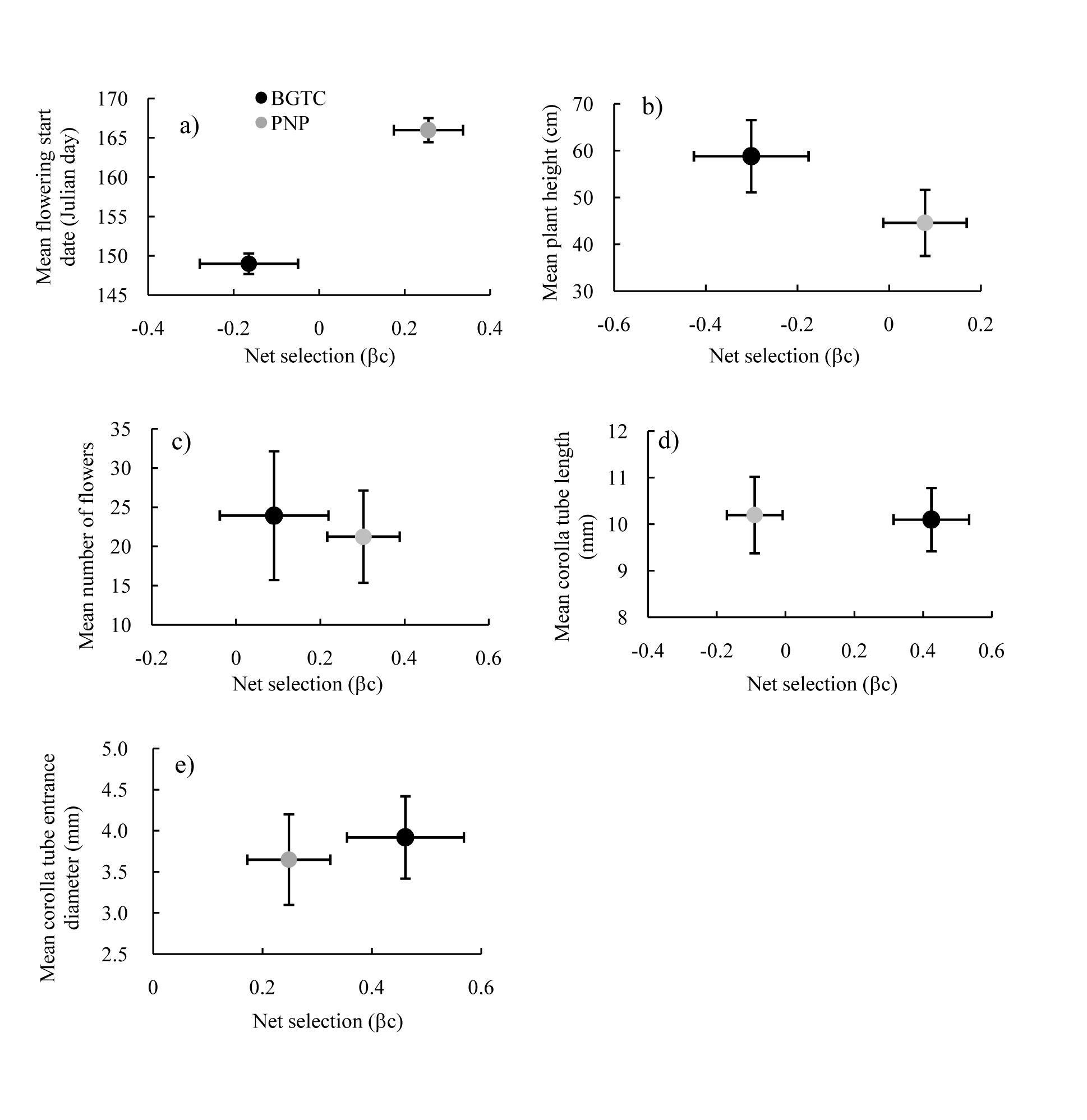

Supplement: Supplementary file 1 [file ECE3-7-7599-s001.jpg]
