# Supplementary material for: Phenotypic selection on flowering phenology and pollination efficiency traits between Primula populations with different pollinator assemblages
Source: Ecol Evol. 2017 Aug 17;7(19):7599–608. doi: 10.1002/ece3.3258 (PMC5632619; doi:10.1002/ece3.3258)

**SUPPORTING INFORMATION**

**TABLE S1** Differences of floral traits and reproductive performance among L- and S-morph plants between two *Primula secundiflora* populations.

**TABLE S2** The effect of population and pollination treatment (open-pollinated control vs. supplemental hand pollination) on plant traits and plant performance analysed with two-way ANOVA.

**TABLE S3** Phenotypic correlations among traits by pollination treatment within the two *P. secundiflora* populations.

**TABLE S4** *P-*values from ANCOVAs testing for differences in net directional selection and pollinator-mediated selection between populations and between pollinations in *P. secundiflora*.

**FIGURE S1** Relationship between net directional selection (β_C_±SE) and mean trait value (±SE) in the two *P. secundiflora* populations.

**TABLE S1 Differences of floral traits and reproductive performance among L- and S-morph plants at two *Primula secundiflora* populations. These were analyses with one-way ANOVA. Bold *P*-values indicate significant effects (at the significance level of 0.05).**

| Traits | BGTC population | | | | |  | PNP population | | | | |
| --- | --- | --- | --- | --- | --- | --- | --- | --- | --- | --- | --- |
|  | Open-pollinated | |  | Supplemental hand pollination | |  | Open-pollinated | |  | Supplemental hand pollination | |
|  | *F_1,65_* | *P* |  | *F_1,70_* | *P* |  | *F_1,63_* | *P* |  | *F_1,55_* | *P* |
| Flowering start date | 0.113 | 0.738 |  | 0.005 | 0.943 |  | 0.153 | 0.697 |  | 0.494 | 0.485 |
| Plant height | 0.01 | 0.921 |  | 0.525 | 0.471 |  | 0.001 | 0.979 |  | 3.449 | 0.069 |
| Number of flowers | 1.111 | 0.296 |  | 0.293 | 0.59 |  | 2.857 | 0.096 |  | 2.858 | 0.097 |
| Corolla tube length | 3.145 | 0.087 |  | 2.987 | 0.166 |  | 2.855 | 0.096 |  | 0.316 | 0.357 |
| Corolla tube entrance diameter | 2.111 | 0.179 |  | 2.445 | 0.161 |  | 2.372 | 0.14 |  | 1.125 | 0.259 |
| Female fitness  (total viable seeds per individual) | 0.656 | 0.421 |  | 2.237 | 0.139 |  | 1.894 | 0.322 |  | 2.234 | 0.141 |
| Number of fruits | 0.001 | 0.979 |  | 2.055 | 0.156 |  | **6.86** | **0.022** |  | 1.798 | 0.186 |
| Viable seeds per fruit | 0.253 | 0.617 |  | 1.685 | 0.199 |  | **4.102** | **0.047** |  | 1.914 | 0.172 |

**TABLE S2** The effect of population and pollination treatment (open-pollinated control vs. supplemental hand pollination) on plant traits and plant performance analysed with two-way ANOVA. Bold *P*-values indicate significant effects (at the significance level of 0.05).

|  | Population | |  | Pollination treatment | |  | Population × Pollination treatment | |
| --- | --- | --- | --- | --- | --- | --- | --- | --- |
| Trait | *F*_1,256_ | *P* |  | *F*_1,256_ | *P* |  | *F*_1,256_ | *P* |
| Flowering start date | 8644.733 | **＜0.001** |  | 1.223 | 0.270 |  | 0.468 | 0.494 |
| Plant height | 325.214 | **＜0.001** |  | 0.179 | 0.672 |  | 4.205 | **0.041** |
| Number of flowers | 48.699 | **＜0.001** |  | 0.141 | 0.708 |  | 23.073 | **＜0.001** |
| Corolla tube length | 0.942 | 0.333 |  | 0.977 | 0.324 |  | 3.396 | 0.067 |
| Corolla tube entrance diameter | 12.829 | **＜0.001** |  | 0.564 | 0.453 |  | 0.454 | 0.501 |
| Number of fruits | 21.756 | **＜0.001** |  | 73.938 | **＜0.001** |  | 24.193 | **＜0.001** |
| Viable seeds per fruit | 33.798 | **＜0.001** |  | 120.693 | **＜0.001** |  | 70.149 | **＜0.001** |
| Female fitness (total viable seeds per individual) | 38.021 | **＜0.001** |  | 131.855 | **＜0.001** |  | 55.776 | **＜0.001** |

**TABLE S3** Phenotypic correlations among traits by pollination treatment within the two *P. secundiflora* populations. C, open-pollinated control; HP, supplemental hand pollination. *, *P*＜0.05; **, *P*＜0.01.

**BGTC**

C and HP treatment (C above the diagonal, HP below the diagonal)

| Trait | Flowering start date | Plant height | Number of flowers | Corolla tube length | Corolla tube entrance diameter |
| --- | --- | --- | --- | --- | --- |
| Flowering start date |  | -0.250^*^ | -0.052 | -0.160 | 0.100 |
| Plant height | -0.274^*^ |  | 0.303^*^ | 0.129 | 0.126 |
| Number of flowers | -0.065 | -0.012 |  | 0.023 | -0.003 |
| Corolla tube length | -0.095 | 0.074 | 0.066 |  | -0.424^**^ |
| Corolla tube entrance diameter | 0.030 | -0.115 | -0.021 | -0.587^**^ |  |

**PNP**

C and HP treatment (C above the diagonal, HP below the diagonal)

| Trait | Flowering start date | Plant height | Number of flowers | Corolla tube length | Corolla tube entrance diameter |
| --- | --- | --- | --- | --- | --- |
| Flowering start date |  | -0.191 | -0.012 | -0.026 | -0.147 |
| Plant height | 0.058 |  | 0.445^**^ | 0.268^*^ | 0.091 |
| Number of flowers | 0.091 | 0.306^*^ |  | -0.147 | 0.166 |
| Corolla tube length | 0.065 | 0.228 | 0.025 |  | -0.356^**^ |
| Corolla tube entrance diameter | 0.146 | -0.116 | 0.231 | -0.606^**^ |  |

**TABLE S4** *P-*values from ANCOVAs testing for differences in net directional selection and pollinator-mediated selection between populations and between pollinations in *P. secundiflora*.

|  | Net directional selection varied between populations (n=130) | | | | | |  | | Pollinator-mediated selection varied between populations (n=257) | | | | | | | | | | | |
| --- | --- | --- | --- | --- | --- | --- | --- | --- | --- | --- | --- | --- | --- | --- | --- | --- | --- | --- | --- | --- |
| Trait | *P* trait | | *P* population | | *P* trait × population | |  | | *P* trait | | *P* pollination | | *P* population | | *P* trait × pollination | | *P* trait × population | | *P* trait × pollination × population | |
| Flowering start date | 0.961 | 0.995 | | **0.026** | |  | | 0.516 | | 0.994 | | 0.994 | | 0.964 | | **0.042** | | 0.178 | |  |
| Plant height | 0.125 | 0.995 | | 0.111 | |  | | 0.208 | | 0.994 | | 0.994 | | 0.803 | | 0.085 | | 0.287 | |  |
| Number of flowers | **＜0.001** | 0.995 | | **0.050** | |  | | **＜0.001** | | 0.994 | | 0.994 | | 0.889 | | 0.398 | | **0.034** | |  |
| Corolla tube length | 0.611 | 0.995 | | **0.029** | |  | | 0.897 | | 0.994 | | 0.994 | | 0.399 | | **0.030** | | 0.084 | |  |
| Corolla tube entrance diameter | **＜0.001** | 0.995 | | 0.104 | |  | | **＜0.001** | | 0.994 | | 0.994 | | **0.003** | | **0.014** | | 0.640 | |  |

**FIGURE S1** Relationship between net directional selection (β_C_±SE) and mean trait value (±SE) in the two *P. secundiflora* populations. a, flowering start date; b, plant height; c, number of flowers; d, corolla tube length; e, corolla tube entrance diameter.


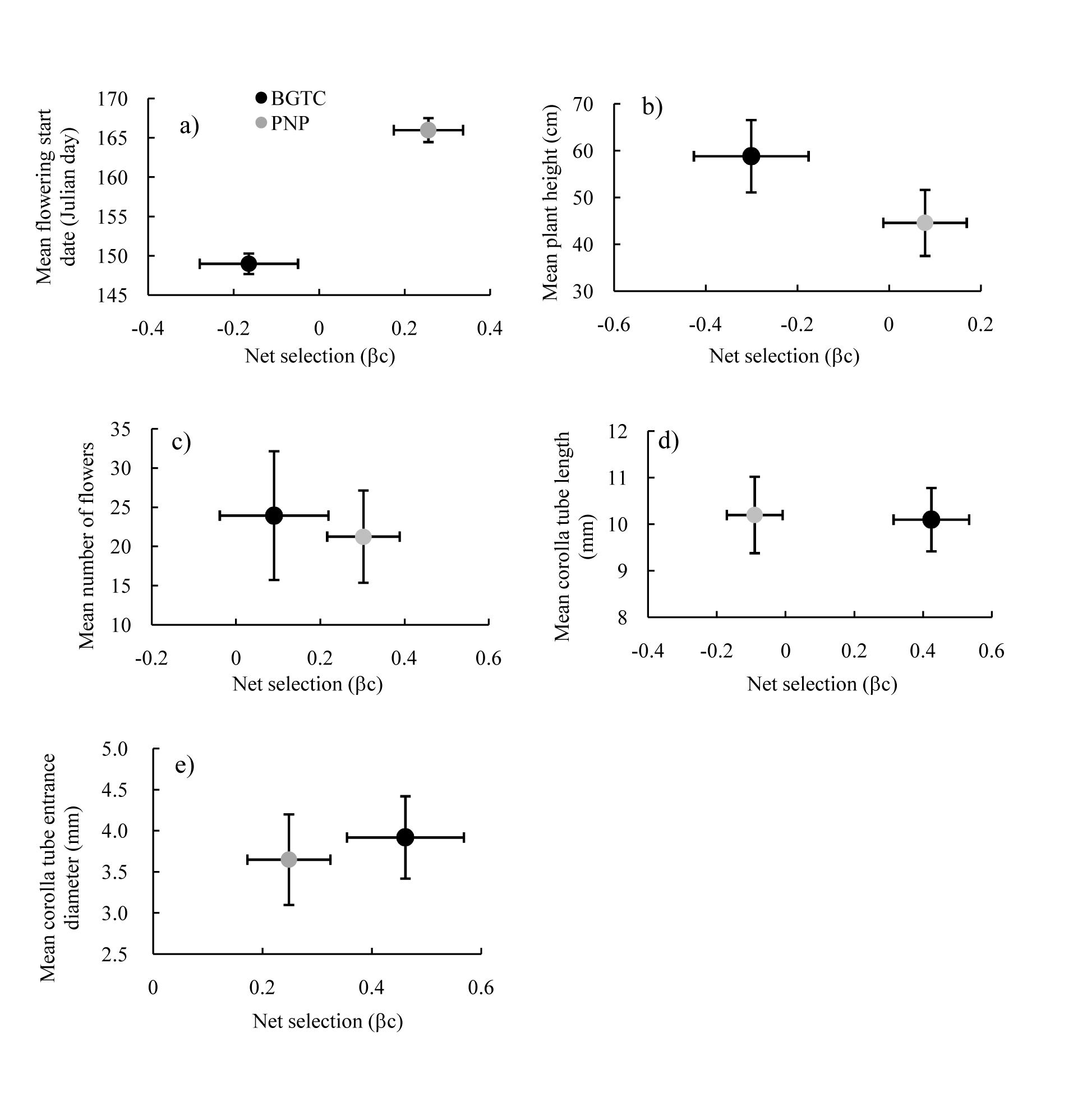

Supplement: Supplementary file 2 [file ECE3-7-7599-s002.doc]
